# Robotic Ventral Transabdominal Preperitoneal Repair of Uncomplicated Spigelian Hernia

**DOI:** 10.7759/cureus.34441

**Published:** 2023-01-31

**Authors:** Boluwatito T Abraham, Hussein Sheikhaden, Jae Woo Lee, John T Williams

**Affiliations:** 1 General Surgery, Trinity School of Medicine, Warner Robins, USA; 2 General Surgery, Trinity School of Medicine, Macon, USA; 3 General Surgery, Coliseum Medical Centers, Macon, USA

**Keywords:** periumbilical hernia, hybrid mesh, hernia, robotic intraperitoneal onlay mesh (r-ipom), robotic ventral transabdominal preperitoneal (r-vtapp), robotic hernia repair, transabdominal preperitoneal (tapp), spigelian hernia

## Abstract

Spigelian hernias are rare herniations through the Spigelian fascia, with an incidence rate of 0.12-2.0% of all hernias. Diagnosis may be difficult due to a potential lack of symptoms until complications arise. Therefore, imaging with either ultrasound or CT with oral contrast is recommended to confirm the diagnosis if a Spigelian hernia is suspected. Once the diagnosis has been established, it is essential that operative repair be performed as soon as possible because 24% of Spigelian hernias become incarcerated, and 27% of Spigelian hernias lead to strangulation. Management options include open surgery, laparoscopic surgery, and robotic surgery. This case report discusses the management of a 47-year-old man with an uncomplicated Spigelian hernia that was repaired with the robotic ventral transabdominal preperitoneal repair technique.

## Introduction

A Spigelian hernia is defined as the herniation of abdominal contents or peritoneum through a defect in the Spigelian fascia, which consists of the combined aponeuroses of the transversus abdominis and internal oblique muscles [1]. With an incidence rate of 0.12-2.0% of all abdominal hernias, Spigelian hernias can be difficult to recognize [1]. However, due to their incarceration rate of 24% and their strangulation rate of 27%, healthcare providers need to be aware of how these patients typically present and the best course of management for them [2, 1]. We report the case of a Spigelian hernia repair without obstruction or gangrene.

## Case presentation

**Figure 1:**
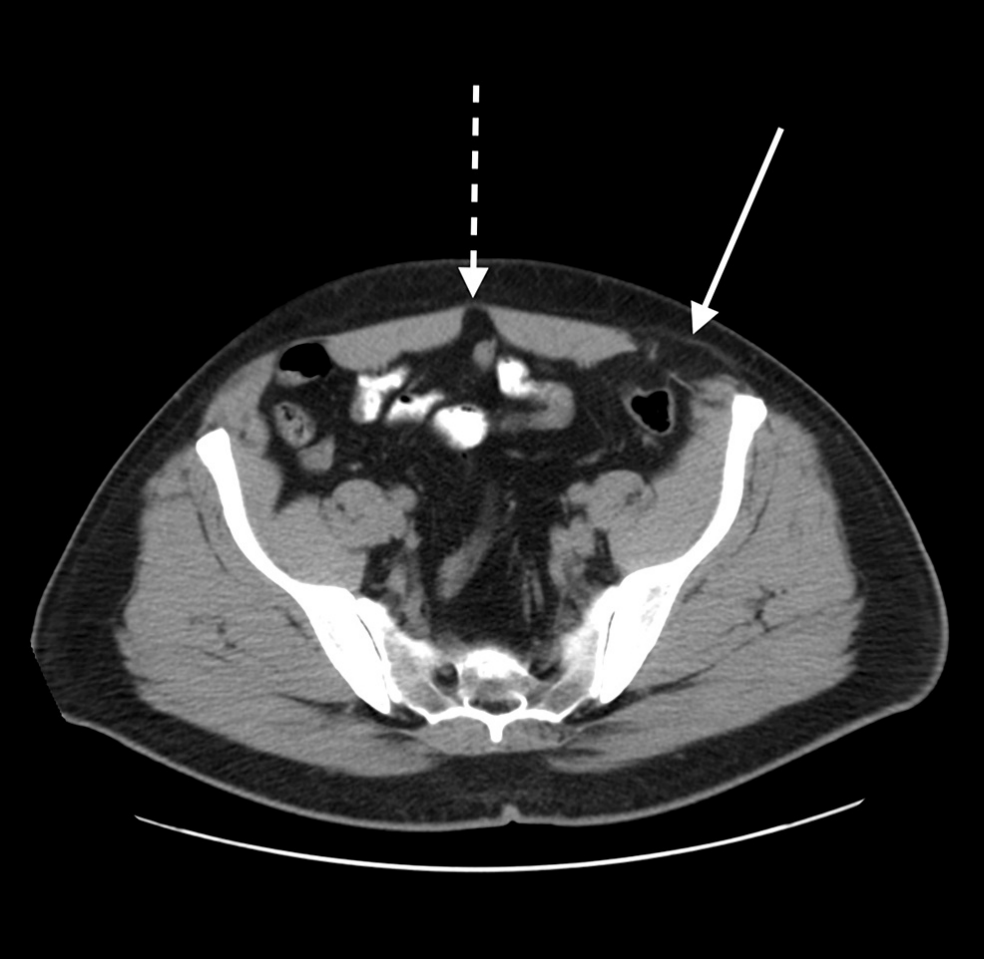
CT scan of the abdomen and pelvis without contrast, which displays a periumbilical incisional hernia (dotted arrow) and a left-sided Spigelian hernia (plain arrow).

A 47-year-old man presented with several months of left lower abdominal quadrant discomfort and swelling, which was exacerbated by straining and alleviated with rest. When these symptoms initially began, the patient stated that he had first sneezed, then felt a tearing sensation in his left lower abdominal quadrant, and finally noticed a bulge in his left lower abdominal quadrant. He denied any nausea, vomiting, fever, chills, sweating, or changes in bowel movements. Past medical history was significant for hiatal hernia, right inguinal hernia, left inguinal hernia, hypercholesterolemia, and acid reflux. The patient was a non-smoker and only consumed alcohol occasionally. On examination, the patient was alert and oriented without tachycardia or tachypnea. No masses were palpated during the abdominal exam; however, the patient did describe a vague sensation of fullness below the level of the umbilicus in the left lower abdominal quadrant at the semilunar line. Additionally, there was no evidence of a recurrent bilateral inguinal hernia. Upon further investigation with CT imaging, a 3.5 cm left-sided Spigelian hernia and a 3 cm periumbilical incisional hernia were identified, as seen in Figure 1. Robotic surgery was then scheduled with the consent of the patient.

**Figure 2:**
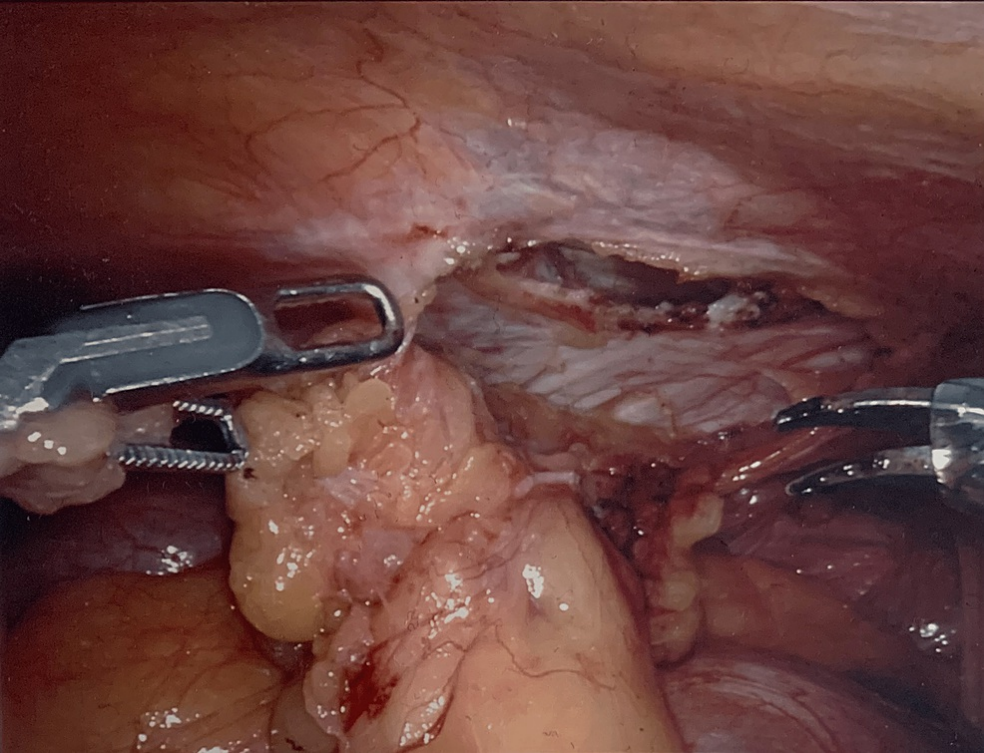
Fat removal from periumbilical defect.

**Figure 3:**
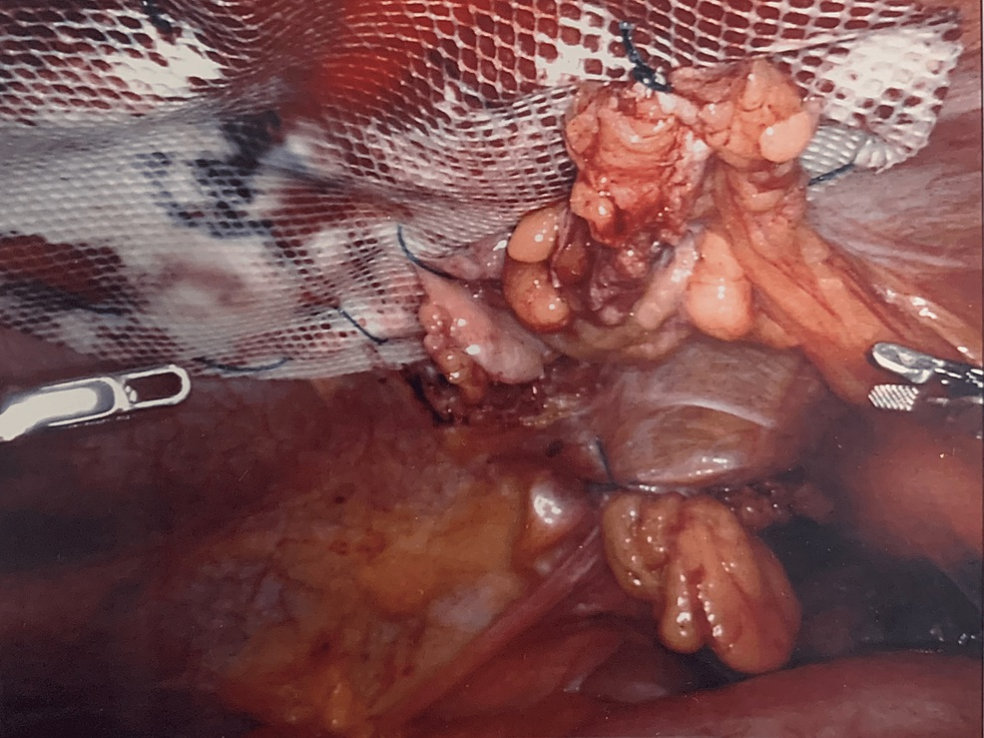
GORE® SYNECOR hybrid composite mesh sutured over periumbilical defect intraperitoneally.

**Figure 4:**
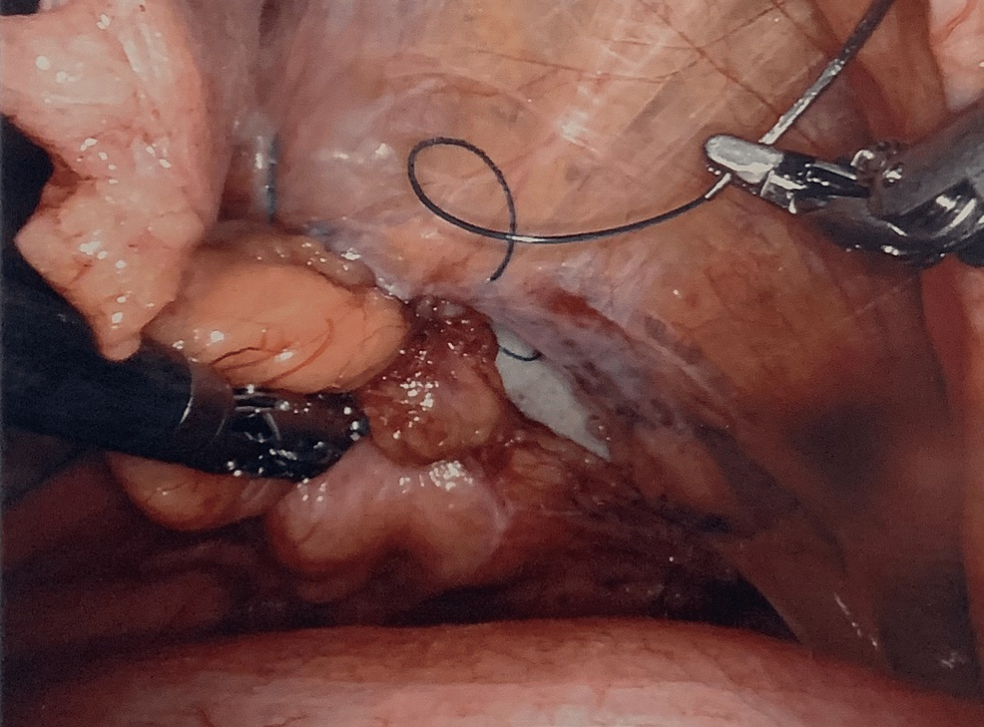
Synthetic absorbable mesh sutured over Spigelian defect preperitoneally.

After the general anesthesia team had performed an initial tap block, they prepped and draped the patient’s abdomen and then intubated the patient. Timeout was then followed. First, a skin incision was made in the left upper abdominal quadrant, in which a Veress needle was inserted and used to establish pneumoperitoneum, along with an optical viewing trochar. Next, four robotic trochars were inserted in the patient’s right abdomen. Afterward, the Da Vinci robotic surgical system was docked. The initial step of the robotic surgery was used to dissect the periumbilical incisional hernia, reduce its contents, and pull the fat out of the defect. This left the preperitoneal space with an opening, as seen in Figure 2. Subsequently, the periumbilical incisional hernia defect was closed intraperitoneally with 0 V-Loc sutures and a 12 cm circle GORE® SYNECOR hybrid composite mesh, as seen in Figure 3. Next, the left-sided Spigelian hernia defect was closed preperitoneally with 0 V-Loc sutures and a 9 cm preperitoneal synthetic absorbable mesh, as seen in Figure 4. Two separate meshes were used during the course of the surgery because the distance between the periumbilical incisional hernia defect and the left-sided Spigelian hernia defect was too far for a single mesh to encompass. Furthermore, a GORE® SYNECOR hybrid composite mesh was used to close the periumbilical incisional hernia defect intraperitoneally because it has a nonporous polyglycolic acid/trimethylene carbonate (PGA:TMC) film layer that reduces the risk of intraperitoneal adhesions. However, this nonporous PGA:TMC film layer was not needed for the left-sided Spigelian hernia that was closed preperitoneally.

After both hernia defects had been closed, the sigmoid colon was pulled to the side in order to explore the pelvic cavity for the location of a suspected pelvic cyst. However, a location where fluid could have accumulated to create a pelvic cyst could not be found, and it was eventually determined that the pelvic cyst had probably been marsupialized. Therefore, it was decided that repeat imaging could be conducted in the future. Lastly, the pneumoperitoneum was released, and all incisions were closed with 4-0 Vicryl sutures and Dermabond. The patient was then taken to the recovery room in stable condition, and his postoperative recovery period was unremarkable and without complications.

## Discussion

Spigelian hernias were first defined by Flemish anatomist Josef Klinkosch in 1764 and named after Belgian anatomist Adriaan van den Spiegel who defined the semilunar line in 1627 [3]. Spigelian hernias typically occur due to increased intra-abdominal pressure, abdominal trauma, or weakening of the abdominal aponeurotic layers [1]. Therefore, risk factors for Spigelian hernias include previous abdominal operations, straining from bladder outlet obstruction, chronic obstructive pulmonary disease, chronic cough, ascites, multiple pregnancies, obesity, collagen disorders, and aging [4, 1]. Spigelian hernias are most common on the left side, in patients between ages 40 and 70 years old and in women; however, they have been noted to occasionally occur in male children with undescended testes [5]. Furthermore, they are uncommonly superior to the umbilicus due to the extension of the transversus abdominus muscle and internal oblique muscle into the posterior rectus sheath, which strengthens the Spigelian fascia in the upper abdominal quadrants [6]. 90% of Spigelian hernias occur below the umbilicus, 0-6 cm cephalad to the interspinous plane, where the spigelian aponeurosis is the widest and most weak [4]. Contents have been known to include the small intestine, omentum, sigmoid colon, cecum, and appendix [2].

The typical presentation of a Spigelian hernia is an intermittently palpable bulge along the semilunar line, which is most prominent when standing or during Valsalva maneuvers [1, 7]. However, Spigelian hernias may evade clinical detection during a physical exam if they remain deep in the musculature of the external oblique or if the patient is obese [3]. This leads to a 50% detection rate of Spigelian hernias by clinicians when based on a physical exam alone [5, 1]. Therefore, evaluation with ultrasound is recommended as first-line imaging if there is high clinical suspicion; followed by CT imaging with oral contrast to discern whether any contents are included within the hernia [5]. Ultrasound has a sensitivity of 90% and a positive predictive value (PPV) of 100%, while the sensitivity and PPV for CT are both 100% [4].

Oftentimes, a Spigelian hernia is not suspected until the patient experiences pre-operative complications, such as incarceration, strangulation, or bowel obstruction [1]. Compared to other types of hernias, Spigelian hernias display an incarceration rate of 24% and a strangulation rate of 27% due to the rigid fascia surrounding the hernia [2, 5]. Therefore, if a physical exam reveals signs or symptoms of strangulation, an irreducible hernia with overlying erythema and tenderness, rebound tenderness, involuntary guarding, or rigidity, then it is essential to schedule operative repair of the hernia as soon as possible [1]. If operative repair of a Spigelian hernia is done quickly, the prognosis is typically excellent, particularly if the hernia is uncomplicated [1]. Operative repair of Spigelian hernias can be done openly, laparoscopically, or robotically. The conventional open surgery technique utilizes a transverse Gridiron’s incision centered on McBurney’s point, followed by a herniotomy, and then closes the hernia defect with a mesh [1]. Three major laparoscopic techniques can be used for Spigelian hernias. The Intraperitoneal Onlay Mesh (IPOM) repair technique first reduces the hernia and then places a mesh posterior to the peritoneum, with at least 5 cm of circumferential margin beyond the edges of the hernia defect [1]. The Transabdominal Preperitoneal (TAPP) repair technique reduces the hernia, dissects the peritoneum in order to create peritoneal flaps, and then places the mesh anterior to the peritoneum with a similar circumferential margin as the IPOM technique [1]. Lastly, the Total Extraperitoneal (TEP) repair technique inflates a balloon in order to allow for extraperitoneal hernia reduction without ever entering into the peritoneum [1]. When compared to open surgery, it was found that both TEP and IPOM significantly reduce patient morbidity and the length of hospital stay [8, 9]. Robotic repairs of Spigelian hernias are similar to laparoscopic techniques. However, they grant better access to the different layers of the abdominal wall than conventional laparoscopy. They also have the benefits of better ergonomics, greater freedom of instrumentation, and increased image stability [10]. This allows robotic repairs to integrate the major advantages of open procedures together with those of laparoscopic procedures, resulting in a lower hernia recurrence rate than laparoscopic procedures and a lower hernia complication rate than open procedures [10]. The techniques include robotic intraperitoneal onlay mesh (r-IPOM), robotic-enhanced view total extraperitoneal plasty (r-eTEP), robotic ventral TAPP (r-vTAPP), robotic transversus abdominis release (r-TAR), and robotic transabdominal retromuscular umbilical prosthetic hernia repair (TARUP) [11]. While r-IPOM, r-eTEP, and r-vTAPP share a similar approach to the three major laparoscopic techniques but with robotic assistance, r-TAR creates an immense retro-muscular plane and allows bilaminar ingrowth of the mesh, allowing primary closure of defect [12, 13]. In contrast, TARUP inserts the retro-muscular mesh after first opening the ipsilateral posterior rectus fascia [14]. Typically, r-vTAPP is indicated for primary ventral hernias <4 cm, r-TARUP is indicated for primary ventral hernias >4 cm, for incisional hernias <7 cm, and r-TAR is indicated for incisional hernias >8 cm [10].

Postoperative complications from robotic repair of Spigelian hernias occur at a rate of 12%. They can include mesh infections, injuries to the abdominal viscera, seromas, hematomas, surgical site infections, and hernia reoccurrence [1, 11]. Appropriate mesh selection is an essential step of hernia repairs that can affect the rate of hernia recurrence and the rate of infections, hematomas, and seromas [15]. Therefore, surgeons must understand the advantages and disadvantages that different mesh materials may present. The meshes used for hernia repair can be classified as synthetic, biological, or a hybridization of both. Permanent synthetic meshes consist of polypropylene, polytetrafluoroethylene (PTFE), or polyester, whereas absorbable synthetic meshes consist of dexon or vicryl [16]. Generally, permanent synthetic meshes are more susceptible to infection than absorbable synthetic meshes; nonetheless, permanent synthetic meshes tend to have a lower rate of hernia recurrence than absorbable synthetic meshes [16]. Biological meshes consist of the collagen matrix of human, bovine, or porcine tissues that have been decellularized. They offer the benefit of a lower foreign body response than synthetic meshes and a higher resistance to infection [16]. Hybrid meshes attempt to combine the strengths of synthetic and biological meshes. In this surgery, a GORE® SYNECOR hybrid composite mesh was first used to close the periumbilical incisional hernia defect intraperitoneally. Then a synthetic absorbable preperitoneal mesh was used to close the left-sided Spigelian hernia. The GORE® SYNECOR hybrid composite mesh consists of a bioabsorbable 3D web scaffold layer, a PTFE layer, and a nonporous PGA:TMC film layer [15]. The bioabsorbable 3D web scaffold layer allows for tissue ingrowth while reducing the risk of infection in the PTFE layer. The PTFE layer offers the benefit of high durability, and the nonporous PGA:TMC film layer reduces the risk of adhesions when a mesh is placed intraperitoneally [15, 16]. These mesh features help ensure greater quality of life for a patient postoperatively.

## Conclusions

Due to the low incidence rate of Spigelian hernias, clinicians need to be aware of their typical presentation as an intermittent palpable bulge along the semilunar line, which is most prominent with standing or during Valsalva maneuvers. Once a Spigelian hernia has been confirmed with ultrasound imaging followed by CT imaging with oral contrast, it is essential to schedule an operative repair as soon as possible due to their 24% incarceration rate and 27% strangulation rate. If a Spigelian hernia patient undergoes surgery in a timely manner, then they are likely to experience better outcomes. Amongst the surgical modes available, robotic surgery offers the benefits of improved access to the different abdominal wall layers, better ergonomics, greater freedom of instrumentation, and increased image stability. Furthermore, robotic surgery should be considered for Spigelian hernia patients due to a lower hernia recurrence rate than laparoscopic procedures and a lower hernia complication rate than open procedures. Lastly, when repairing a hernia that is at risk of intraperitoneal adhesions, surgeons should consider the use a hybrid composite mesh that offers the benefits of high durability, low infection rate, and a low rate of adhesions.

Learning objectives include (I) Recognizing the typical presentation of a Spigelian hernia and the associated risk factors; II) Acknowledging the high rate of complications resulting from Spigelian hernias, despite their low rate of incidence; III) Discerning the appropriate imaging modalities to be used for Spigelian hernias; IV) Comparing and contrasting the advantages of different surgical techniques that can be used to repair a Spigelian hernia; and V) Comparing and contrasting the advantages of different mesh materials.
